# Smoking among Malaysian adults aged ≥15 years: A secondary dataset analysis of Global Adult Tobacco Survey-Malaysia 2023 (GATS-M 2023)

**DOI:** 10.18332/tid/211250

**Published:** 2025-11-26

**Authors:** Kuang Hock Lim, Yoon Ling Cheong, Jia Hui Lim, Sumarni Mohd Ghazali, Kee Chee Cheong, Chien Huey Teh, Pei Pei Heng, Ali Aman Marine, Yong Kang Cheah, Nor Syahaliyana Saidin, Mohd Hazilas Mat Hashim, Hui Li Lim

**Affiliations:** 1 Independent Researcher, Seri Kembangan, Malaysia; 2 Institute for Medical Research, National Institutes of Health, Ministry of Health Malaysia, Shah Alam, Malaysia; 3 School of Pharmacy, Faculty of Health and Medical Sciences, Taylor’s University, Subang Jaya, Malaysia; 4 Biostatistics and Data Repository Sector, National Institutes of Health, Ministry of Health Malaysia, Shah Alam, Malaysia; 5 Institute for Public Health, National Institutes of Health, Ministry of Health Malaysia, Shah Alam, Malaysia; 6 School of Economics, Finance and Banking, University Utara Malaysia, Sintok, Malaysia; 7 Institute for Clinical Research, National Institutes of Health, Ministry of Health Malaysia, Shah Alam, Malaysia

**Keywords:** smoking, Malaysian adults, sociodemographic factors, GATS-M

## Abstract

**INTRODUCTION:**

The ongoing assessment of smoking rates and their related factors is a crucial component of anti-smoking initiatives and is essential for evaluating the success of anti-smoking strategies and policies. This research aimed to ascertain the prevalence of smoking and to identify the sociodemographic factors linked to smoking among adults in Malaysia aged ≥15 years.

**METHODS:**

We analyzed the secondary data of GATS-M 2023, which employed a cross-sectional design with a representative sample of 4269 adults in Malaysia aged ≥15 years, selected through a stratified, two-stage proportionate-to-size sampling technique. The research team collected the GATS-M data through face-to-face interviews conducted by trained research assistants, using a standardized, validated questionnaire. A multivariable logistic regression analysis was performed to identify sociodemographic factors associated with smoking among Malaysians.

**RESULTS:**

The overall smoking prevalence was found to be 19.0% (95% CI: 17.1–21.1), with males exhibiting a significantly higher prevalence than females (35.7%; 95% CI: 32.0–39.5 vs 1.5%; 95% CI: 0.8–3.1). The highest rates of smoking were noted among individuals of other ethnic backgrounds (39.1%), those aged 25–44 years (24.9%), and individuals who completed primary school but less than secondary school 95% CI: 2.60–5.91 (23.6%). Multivariable analysis revealed that Males from Malay (AOR=3.92; 95% CI: 2.60–5.91), Indian (AOR=3.17; 95% CI: 1.50–3.74), Other Bumiputra (AOR=3.14; 95% CI: 1.83–0.33), and other ethnic groups (AOR=4.77; 95% CI: 2.36–9.65) (Chinese ethnic as reference), and individuals with primary (AOR=2.98; 95% CI: 1.81–4.90) and secondary education level, showed a higher risk of being current smokers (AOR=1.81; 95% CI: 1.17–2.80, tertiary education level as reference) whilst no similar trends were found among female adults.

**CONCLUSIONS:**

The smoking prevalence among Malaysian adults aged ≥15 years showed a slight decrease. There is a need for more anti-smoking policies or interventions, particularly aimed at males, Malays, younger adults, and those with lower levels of education, to further reduce the smoking prevalence in Malaysia.

## INTRODUCTION

The tobacco epidemic represents one of the most significant public health challenges that the world has encountered, leading to over 7 million deaths each year, along with disability and prolonged suffering due to tobacco-related illnesses^1^. Smoking-related illnesses, including cancer and cardiovascular diseases, are the leading cause of premature mortality worldwide^1^. Approximately 80% of the 1.3 billion tobacco users around the globe reside in low- and middle-income nations^1,2^ where the impact of tobacco-related diseases and fatalities is most severe. Tobacco consumption exacerbates poverty by reallocating household expenditures from essential needs such as food and shelter towards tobacco products^3,4^. This spending pattern is challenging to change due to the addictive nature of smoking. The financial implications of tobacco use are considerable, encompassing substantial healthcare expenses for treating diseases linked to tobacco consumption^3,4^, in addition to the loss of human capital that arises from tobacco-related health issues. The total healthcare spending attributed to diseases caused by smoking reached a purchasing power parity (PPP) of $467 billion (approximately $422 billion), representing 5.7% of global health expenditures. Nearly 40% of this financial burden was borne by developing nations^5^, underscoring the significant challenges these countries face. In Malaysia, smoking-related illnesses have been the foremost cause of death for the last thirty years^6^. The latest burden of disease study in Malaysia revealed that morbidity and mortality due to smoking contributed substantially to the burden of disease in Malaysia. The primary cause of premature death among Malaysians is ischemic heart disease, responsible for 17.7% of cases, cerebrovascular disease (stroke) at 8.0%, and diabetes mellitus at 3.9%^7^.

The Malaysian government has taken a proactive anti-smoking initiative to reduce the burden of smoking by ratifying the World Health Organization Framework Convention on Tobacco Control in 2005^8^, introducing laws to regulate the sale and procurement, and implementing smoke-free zones – enforcing anti-smoking laws^9^. Additionally, smoking cessation programs have been strengthened by providing smoking cessation services in public healthcare facilities and by collaborating with private healthcare providers. Furthermore, increasing the price of tobacco products and promoting health activities in schools^10^, in collaboration with the Ministry of Education, Malaysia, aim to reduce the incidence of smoking among youth in Malaysia. In addition, the Ministry of Health, Malaysia, also carried out frequent national health and morbidity surveys and reduced each cycle of surveys from once every ten years to every 4 years to ensure the latest data for the formulation of suitable measures to increase healthcare for the Malaysian population, including health-related behavior such as smoking. All the measures implemented were in line with the MPOWER approach outlined in the FCTC Convention^8^.

In 2019, the smoking prevalence was 21.3% (95% CI: 19.9–22.8). The researchers’ study found the rate was consistently elevated among males (40.5–43.9%), adults aged 25–44 years (25.4–29.0%), Malays (22.6–24.7%), individuals from other ethnic backgrounds (30.0–35.0%), and the self-employed population (33.7–44.6%). A multiple logistic regression analysis indicated that the adjusted odds ratio (AOR) for smoking was greater among males, younger and middle-aged individuals, Malays, and those with lower levels of education^11^. However, the introduction of tobacco products such as e-cigarettes and heated tobacco into the market may change the pattern of tobacco product use among the Malaysian population; in addition, the changes in social demographics among the Malaysian population and the effect of anti-smoking measures implemented might change the landscape of smoking among Malaysians. The latest information is crucial for formulating effective policies to strengthen anti-smoking initiatives. This study aims to describe the prevalence, smoking initiation, and factors associated with smoking among adults aged ≥15 years in Malaysia using the GATS-M 2023 data.

## METHODS

The GATS-M 2023 employed a cross-sectional study design with a multi-stage, geographically clustered sampling approach to generate data representative at the national level. The survey targeted individuals aged ≥15 years, with Malaysia’s urban and rural areas forming the first stratum. Enumeration blocks (EBs), defined by the Department of Statistics as areas containing 80–120 living quarters (LQs) based on the 2020 census, served as the primary sampling unit; EBs were randomly selected based on proportionate to population size, while LQs were the secondary unit. At the third stage, the researcher team chose one household member aged ≥15 years from each chosen LQ. In total, 426 EBs (222 urban, 204 rural) and 5112 LQs were sampled. The survey was carried out by the Institute for Public Health in partnership with the Disease Control Division of the Ministry of Health Malaysia. A sample of 5780 households was selected, and the research team chose one individual randomly from each participating household to participate in the survey. The collection of survey data was conducted electronically using handheld devices. Ultimately, 4269 individual interviews were completed, resulting in an overall response rate of 81.5%. We analyze the secondary data from the GATS-M conducted in June and July 2023.

The GATS-M adopted a validated tobacco survey questionnaire, which had been validated in the GATS-M 2011^12^, NHMS 2015 studies^13^, and NHMS 2019^11^, with slight modifications in line with the current development of smoking research, including items encompassing the latest novel tobacco products on the market, such as heated tobacco products. These instruments include inquiries regarding the smoking status and types of tobacco products, the use of smokeless tobacco, e-cigarette usage, exposure to secondhand smoke, cessation efforts, anti-cigarette messaging, cigarette advertising, and cigarette purchasing habits. Additionally, the questionnaire gathered information on the respondents’ sociodemographic characteristics, including age, gender, ethnicity, education level, marital status, occupation, and income level. The dependent variable is current smoking, which was measured by the item: ‘Do you currently smoke tobacco daily or less than daily?’. Those who responded ‘Yes’ were classified as ‘current smokers’, whereas individuals who replied ‘Not at all’ were categorized as ‘non-smokers’.

The independent variables were residence (urban, rural), sex (male, female), age group (15–24; 25–44; 45–64; ≥65 years), ethnicity (Malay, Chinese, Indian, Other Bumiputras, Other), education level (no formal education, primary education, secondary education, tertiary education), occupation (government employee, private employee, self-employed, student/retired/homemaker), and marital status (single, married, divorced/separated/widowed).

The data were collected through face-to-face interviews conducted by trained data collectors. Written informed consent was secured from the participants before the interview commenced. The respondents were provided with an explanation of the study, and their participation was entirely voluntary. All information collected will be used solely for research purposes, ensuring the anonymity and confidentiality of the provided information. For participants aged <18 years, consent was obtained from a parent/guardian, along with the participant’s assent.

### Statistical analysis

The dataset underwent cleaning and was weighted based on the sampling design, response rate, and population characteristics of the survey to ensure the findings can be inferred from the target population. We used the chi-squared analysis to examine the relationship between sociodemographic factors and smoking. We included the univariate analysis with a p≤0.25 in the multivariable logistic regression^14^. We carried out an interaction analysis between independent variables and found significant interactions between gender and residence, gender and occupation, and gender and ethnicity. Therefore, two multivariable logistic regressions were carried out, one for each gender. The model’s fit was assessed using a classification table. Data are presented with a 95% confidence level. We conducted all statistical analyses using SPSS version 26^15^, which included the complex sample function.

## RESULTS

A total of 4269 participants took part in GATS-M 2023, with response rate of 81.5%. The distribution of respondents by gender and residence area was nearly balanced. The predominant demographic among the respondents was Malays, with almost two-thirds (64.0%) of respondents being married. One-fifth of respondents attained a tertiary education level, while 70% of respondents were aged 25–64 years (Table 1).

**Table 1:** Sociodemographic characteristics of Malaysian adults, aged ≥15 years, who participated in the Global Adult Tobacco Survey -Malaysia (GATS-M) 2023 (N=4269)

*Characteristics*	*n*	*%*
**Gender**		
Male	2135	50.0
Female	2134	50.0
**Residence**		
Urban	1990	46.6
Rural	2279	53.4
**Ethnicity**		
Malay	2506	58.7
Chinese	602	14.1
Indian	170	4.0
Other Bumiputra	726	16.9
Other	265	6.2
**Age** (years)		
15–24	577	13.5
25–44	1699	39.8
45–64	1328	31.1
≥65	665	15.6
**Education level**		
Less than primary	387	9.1
Completed primary but less than secondary	1128	26.6
Completed secondary	1873	44.2
College and higher	848	20.0
**Marital status**		
Married	2721	64.0
Single	1069	25.1
Divorce/separated/widowed	463	10.9
**Occupation**		
Government	344	8.1
Private	1087	25.5
Self employed	955	24.2
Student/retired/homemaker	1456	34.2
Unemployed	417	9.8

Approximately 4.8 million adults in Malaysia (19.0%) aged ≥15 years were identified as current smokers. The rate of current smokers was notably higher among males (37.5%; 95% CI: 32.0–39.5) in comparison to females (1.5%; 95% CI: 0.8–3.1). The prevalence of smoking was significantly higher among the other ethnic groups (39.1%; 95% CI: 28.7–50.6) when compared to specific ethnic groups. Adults aged 25–44 years (24.9%; 95% CI: 21.8–28.2) exhibited the highest smoking prevalence; however, those with tertiary education (13.4%; 95% CI: 10.5–16.9) showed the lowest smoking prevalence. Conversely, the prevalence of smoking was significantly elevated among self-employed individuals (33.4%; 95% CI: 28.5–38.7) in contrast to private sector employees, government employees, retirees, students, and homemakers (Table 2).

**Table 2:** Prevalence of current smokers among Malaysian adults, aged ≥15 years, who participated in the Global Adult Tobacco Survey–Malaysia (GATS-M) 2023 (N=4269)

*Variables*	*Estimated* *population*	*Current smokers*			
		*n*	*%*	*95% CI*	*p*
**Overall**	4786430	860	19.0	17.1–21.1	
**Gender**					
Male	4598359	834	35.7	32.0–39.5	0.001
Female	188071	26	1.5	0.8–3.1	
**Residence**					
Urban	3569822	348	18.3	15.9–20.9	0.075
Rural	1216608	512	21.7	19.1–24.6	
**Ethnicity**					
Malay	2807888	497	19.7	17.6–21.9	<0.001
Chinese	606854	73	11.5	8.4–15.4	
Indian	372272	29	18.2	11.8–27.1	
Other Bumiputra	426920	166	20.4	16.3–25.2	
Other	564814	94	39.1	28.7–50.6	
**Age** (years)					
15–24	744452	93	12.7	9.5–16.8	<0.001
25–44	2671431	444	24.9	21.8–28.2	
45–64	1147542	250	18.7	15.3–22.5	
≥65	223004	73	9.3	6.7–12.6	
**Education level**					
Less than primary	161154	56	12.2	8.0–18.2	<0.001
Completed primary but less than secondary	1194688	254	23.6	19.6–28.1	
Completed secondary	2410648	423	21.2	18.4–24.3	
College and higher	947937	122	13.4	10.5–16.9	
**Marital status**					
Married	2979863	559	20.6	17.9–23.5	0.009
Single	1659978	244	18.1	15.2–21.4	
Divorce/separated/widowed	127409	54	9.1	6.2–13.0	
**Occupation**					
Government	422049	72	22.9	17.6–29.2	0.001
Private	2149093	330	25.6	21.4–30.2	
Self employed	1585058	330	33.4	28.5–38.7	
Student/retired/homemaker	281950	60	3.4	2.3–5.0	
Unemployed	343659	66	19.1	12.9–27.4	

Rao-Scott chi-squared analysis was used to determine the association between the independent and dependent variables.

The multivariable logistic regression analysis indicated that the probability of smoking among males was considerably higher among the Malays (AOR=3.92; 95% CI: 2.60–5.91) and other ethnic groups (AOR=4.77; 95% CI: 2.36–9.65), exhibiting a greater likelihood of becoming smokers in comparison to different ethnic groups. The highest probability of smoking was among individuals aged 25–44 years (AOR=2.97; 95% CI: 1.76–5.01) and those who completed primary (AOR=1.81; 95% CI: 1.81–4.90) and secondary education (AOR=1.81; 95% CI: 1.17–2.80). Additionally, self-employed individuals (AOR=5.69; 95% CI: 3.33–9.72), private sector employees (AOR=4.87; 95% CI: 2.83–8.37), and government servants (AOR=3.22; 95% CI: 1.73–5.97) also demonstrated increased odds of being smokers compared to students/retired/homemaker. Nevertheless, no significant correlations were found between marital status, residence, and smoking behavior. Among females, no such trend was observed, in which only non-employed respondents showed a higher risk of being current smokers compared to students/retired/homemaker (Table 3).

**Table 3:** Multivariable logistic regression analysis of sociodemographic factors associated with current smoking, stratified by sex, among Malaysian adults, aged ≥15 years, who participated in the Global Adult Tobacco Survey–Malaysia (GATS-M) 2023 (N=4215)

*Variables*	*Male (N=2108)*		*Female (N=2107)*	
	*AOR*	*95% CI*	*AOR*	*95 % CI*
**Residence**				
Urban ®	1		1	
Rural	0.90	0.67–1.21	0.44	0.12–1.66
**Ethnicity**				
Malay	3.92	2.60–5.91	⁋	⁋
Chinese ®	1			
Indian	3.17	1.50–6.74		
Other Bumiputra	3.14	1.85–5.33		
Other	4.77	2.36–9.65		
**Age** (years)				
15–24	1.48	0.82–2.68	1.29	0.72–2.30
25–44	2.97	1.76–5.01	2.10	0.20–21.78
45–64	2.27	1.33–3.87	2.18	0.25–18.75
≥65 ®	1		1	
**Education level**				
Less than primary	1.48	0.68–3.20	0.20	0.02–1.87
Completed primary but less than secondary	2.98	1.81–4.90	0.45	0.06–3.62
Completed secondary	1.81	1.17–2.80	0.86	0.16–4.76
College and higher ®	1		1	
**Marital status**				
Married ®	1		1	
Single	0.82	0.57–1.18	2.51	0.83–7.64
Divorce/separated/widowed	0.73	0.42–1.27	1.60	0.33–7.90
**Occupation**				
Government	3.22	1.73–5.97	3.99	0.57–28.01
Private	4.87	2.83–8.37	1.58	0.18–13.69
Self employed	5.69	3.33–9.72	1.01	0.16–6.48
Student/retired/homemaker ®	1		1	
Unemployed	3.09	1.64–5.82	15.85	2.15–116.81
**Accuracy of prediction of the dependent variable**	83.5%		98.4 %	

AOR: adjusted odds ratio.

⁋ No results available due to the small percentage of current smokers among females.

® Reference categories.

**Figure 1:**
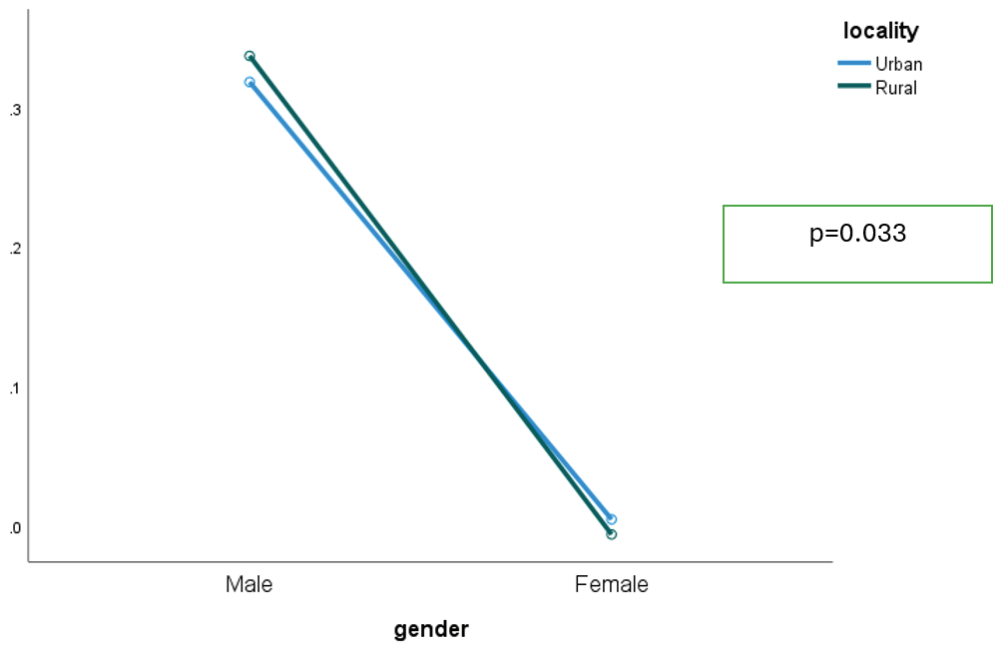
Interaction between gender and residence area among respondents who participated in the GATS-M 2023

Smoking prevalence was markedly higher among males than females in both urban and rural areas. Rural males smoked slightly more than urban males, while among females, prevalence was negligible regardless of residence. The gender gap remained wide across residence, indicating only a weak interaction effect (Figure 1).

**Figure 2:**
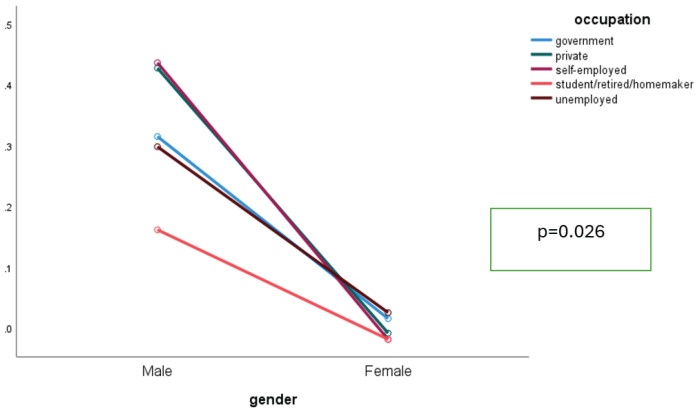
Interaction between gender and type of occupation among respondents who participated in the GATS-M 2023

Figure 2 illustrates the interaction between gender and type of occupation, showing a higher probability of being current smokers among male respondents working in the private sector and self-employed compared to their counterparts in the government sector. Males consistently smoked more than females, but the size of the gender gap varied by occupation. The gap was widest among the self-employed, unemployed, and student/retired/homemaker groups, where female smoking was minimal. In contrast, in the government and private sectors, both sexes smoked more, narrowing the relative difference. This non-parallel pattern indicates an interaction between gender and occupation (Figure 2).

**Figure 3:**
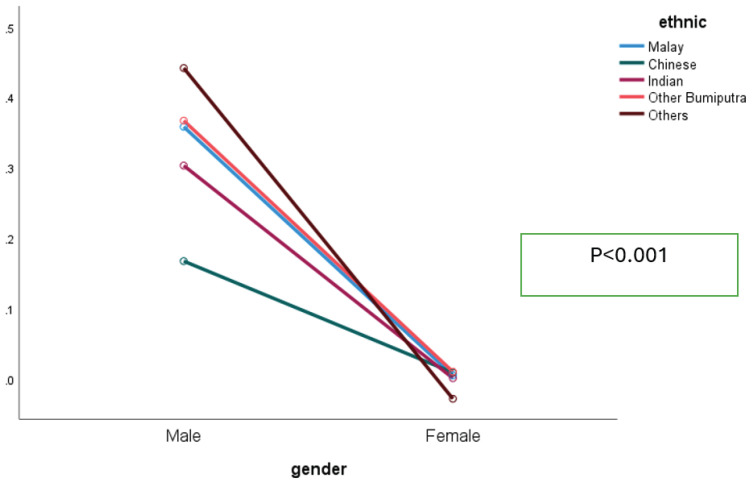
Interaction between gender and ethnicity among respondents who participated in the GATS-M 2023

The interaction between gender and ethnicity showed that males smoked far more than females across all ethnic groups, while female prevalence was negligible. Among males, smoking rates varied by ethnicity – highest among ‘Other’ and ‘Other Bumiputra’, and lowest among Chinese. This indicates a weak interaction: ethnicity shapes smoking mainly among males, but the gender gap remains wide across all groups (Figure 3).

## DISCUSSION

The study found that the prevalence of smoking was 19.0%, which is 2.3% lower than the 21.3% reported by Fadhli et al.^11^ in their findings from the 2019 National Health and Morbidity Survey. A downward trend in smoking prevalence was noted across almost all sociodemographic variables except for Indians, other ethnicities, and government servants, although these changes were not statistically significant. A similar finding was found in a GATS study in the Philippines (from 23.5% in 2015 to 18.4% in 2021)^16^, and the Singapore population Health survey which reported the prevalence of smoking decreased from 10.4% in 2021 to 8.8% by 2023^17^. The findings may be attributed to the implementation of anti-smoking policies in Malaysia. However, the percentage decrease is slight. There is a continued need for the evaluation and enhancement of anti-smoking strategies, implementation, and policies, as the existing trend remains considerably distant from the objective of reducing smoking prevalence to 15% by 2025, as targeted by the Ministry of Health Malaysia^10^.

When compared to other Asian nations, similar smoking prevalence rates were reported in the Philippines (18.5%)^16^, in contrast, China (26.6%)^18^ and Indonesia exhibited a higher rate at 33.5%^19^. Nonetheless, Malaysia’s smoking prevalence remains high in comparison to Singapore’s (8.8%)^17^. These variations may stem from differences in the previous prevalence of smoking in those countries, including socioeconomic factors, cultural norms, tobacco legislation, and taxation policies among the nations. However, our hypotheses need to be investigated in more depth in future studies.

The male-to-female smoker ratio of 23:1 in the current study is lower than NHMS 2019’s (34:1 ratio) previously reported by Fadhli et al.^11^. The ratio is due to the reduction of the prevalence of smoking in males from 40.5% to 35.7% during the four years. The ratio is almost similar to China’s (24:1)^18^. However, the ratio is lower than Vietnam’s (34:1)^20^. Still, it is higher than in the Philippines (9:1)^16^ and Singapore (6.8:1)^17^. The lower incidence of female smokers may be attributed to social norms that discourage female smoking within Malaysian and Asian societies. Asians practice a communal culture that emphasizes a strong focus on collective identity and group harmony. Individuals in these cultures place the needs and objectives of the community above personal desires. They hold in high regard cooperation, interdependence, and the preservation of robust social connections^15^. Therefore, the norm that favors not smoking among females is one of the contributing factors to our findings. The findings in this study and other national studies in Malaysia indicated that the Smoking Epidemic Theory, which posits that the increasing female smoking, along with male smoking at stage 2 and stage 3 smoking among males, began to fall, female smoking caught up^21^, does not apply to the Malaysian population.

The study indicated that males with tertiary education level are less inclined to smoke, which aligns with findings from other studies^17,18^. These findings may be attributed to the enhanced awareness among those with higher education regarding the hazards of cigarette smoking, thereby diminishing the chances of initiating smoking or increasing the probability of cessation among current smokers, as posited by the Health Belief Model and by Protective Motivational Theory^22,23^. Additionally, these individuals may possess superior coping mechanisms for the stressors encountered in daily life^24^, and effective stress management could lower the likelihood of turning to tobacco products for stress relief. Moreover, due to their qualifications, they may be employed as non-manual workers, and a higher responsible position can bring about a positive emotional effect, as it has been shown to lower the risk of smoking^25^. In addition, they might be working in smoke-free environments, which can deter them from starting to smoke or even motivate them to quit smoking. The study also revealed a lower smoking prevalence among those lacking formal education, which is similar to the Fadhli et al.^11^ findings. This finding is particularly unexpected, given the contrasting results reported in previous studies^25,26^. This discrepancy may stem from changes in the characteristics and mindset of individuals without formal education within the population over time. Further investigation is warranted to explore this hypothesis. However, there is no significant association between education level and smoking status among female respondents. The minute proportion of smoking among females may be one of the factors attributed to our findings.

This study found no significant difference in the prevalence and odds of being a smoker by the residence area of respondents. These findings are consistent with the findings of Lim et al.^13^ and Fadhli et al.^11^. However, it contradicts several studies that show that the odds of being a smoker are higher in rural areas. We postulated that the rapid urbanization process might transform rural areas into urban ones, causing a ‘dilution’ of smoking prevalence. These results necessitate a thorough investigation to determine the actual contributing factors.

The current study found a decrease in smoking rates among private employees compared to the findings of Fadhli et al.^11^. However, the prevalence of smoking among government employees increased by around 5% (4.7%). At the same time, it remained static among the self-employed. This finding is surprising considering the increased prevalence of government servants, which may have contributed to the higher exposure to SHS in government departments and health facilities in the GATs-M 2023 study^27^. This finding suggests that the enforcement aspect needs to be strengthened in government departments by enforcement officers, regardless of their status and employment sector. In addition, heads of government departments should establish appropriate mechanisms in the form of a ‘carrot and stick’ within their departments. The decreasing prevalence among private employees is very encouraging. These findings may be attributed to the non-communicable disease intervention (KOSPEN WOW)^28^ activities conducted in the private sector by the Ministry of Health, Malaysia, in conjunction with the increase in smoke-free working areas from 53% to 64%. In this study, most are in the private sector since government offices have been smoke-free areas since 1993^9^, which may be a factor contributing to the findings in this study.

A lower prevalence and lower odds of being a smoker among older people were observed in this study. Several factors may influence this observation. Firstly, the elderly often experience more health issues associated with aging^29^, which may necessitate more frequent visits to healthcare facilities for treatment, thereby indirectly increasing their exposure to anti-smoking messages from health professionals. The guidance provided by health workers is likely to be more readily accepted by older people. Additionally, non-smokers generally enjoy a longer lifespan than smokers^30,31^, as they are typically free from smoking-related health conditions, contributing to the lower smoking prevalence among the older demographic. In addition, a decrease in the prevalence of smoking was also observed among respondents aged 15–24 years. The findings are encouraging, but this finding needs to be interpreted cautiously, as the decrease may be due to respondents in this age group switching to novel tobacco products, such as e-cigarettes, as found in the NHMS: Adolescent Health Survey 2022^32^. However, this needs to be tested in future studies.

No significant association was noted between smoking prevalence and the odds of smoking with marital status. The findings are in contrast to the findings of GATS 2011, in which the adjusted odds ratio (AOR) for smoking was considerably higher among widowers and divorcees compared to married individuals^11^; however, this difference was not significant in the NHMS 2015^13^ and NHMS 2019^11^. Nevertheless, the overall low prevalence of smoking in this demographic can be attributed to the disproportionate representation of female respondents within that group in our study. The significantly reduced prevalence of smoking among females contributed to the overall lower rates in this category. Lim et al.^33^ and Ramsey et al.^34^ indicated that unmarried, divorced, or single adults exhibited higher odds of smoking. Consequently, it seems that the ‘marriage protection’ theory, which posits that married individuals benefit from greater social and psychological support aiding them in quitting smoking, along with the ‘marriage selection’ theory, which suggests that married individuals are more likely to maintain better health by avoiding health risk behaviors such as smoking^35^, do not apply in the context of smoking in Malaysia. The contradictory findings of Lim et al.^32^ highlighted the changes in smoking patterns among Malaysian social demographic characteristics.

A consistent trend of smoking by ethnicity was noted in our study, with a previous National study in Malaysia^11,13^, except for the Indian ethnicity, which showed an increased prevalence of odds of being a current smoker. The prevalence and likelihood of smoking were greater among Malays, ‘Other Bumiputras’, and ‘Other’ ethnicities. These findings align with previous research conducted in Malaysia. In the NHMS and other national studies in Malaysia, the ‘Other Bumiputras’ category primarily consists of ethnic groups from East Malaysia. Non-Malaysian citizens make up the majority of the ‘Other’ ethnic classification. This finding suggests that targeted interventions for Indian ethnic groups, particularly Malay, ‘Other Bumiputra’, and ‘Other’ specific ethnic groups, should be enhanced, taking into account their cultural backgrounds.

Our research indicates that the smoking prevalence among male adults remains significantly high. These results highlight the necessity to reinforce existing anti-smoking policies aimed at male adults, particularly concerning their planning, execution, and assessment. Conversely, the smoking prevalence among female adults in Malaysia continues to be low.

### Strengths and limitations

This study was not without limitations. Firstly, the cross-sectional design of the surveys limits the ability to accurately measure the actual risk of smoking. Secondly, smoking status was assessed through self-reported methods, lacking any biochemical validation, such as cotinine testing in saliva or serum. This procedure may introduce misclassification bias due to the self-reported nature of the initial study. Additionally, we did not account for residual confounding; therefore, the findings may not apply to adults in other countries. The self-reported method is regarded as the standard approach for assessing tobacco use in population studies. Furthermore, the selection of an independent variable that relies exclusively on p-values (p≤0.25) for variable selection may introduce bias or exclude essential confounders.

This study also possesses several strengths. The national surveys featured a large sample size, a robust sampling design, and high response rates across all three surveys, which are acknowledged as strengths in producing reliable results.

## CONCLUSIONS

Our research indicates that the smoking prevalence among male adults in Malaysia remains significantly high. These results highlight the necessity to reinforce existing anti-smoking policies aimed at male adults, particularly concerning their planning, execution, and assessment. Conversely, the smoking prevalence among female adults in Malaysia continues to be low.

## Data Availability

The data supporting this research are available from the authors on reasonable request.
